# Knowledge and Skills Level on Oral Health Among Students at the “Ferdinand I” Military Technical Academy in Bucharest

**DOI:** 10.25122/jml-2020-0099

**Published:** 2020

**Authors:** Ancuta Dumitrita Dan, Doina Lucia Ghergic

**Affiliations:** 1. Titu Maiorescu University, Bucharest, Romania

**Keywords:** Health education, questionnaire, oral health, oral cavity hygiene, young adults

## Abstract

The increase in the number of clinical cases with varying levels of damage to dental structures among young adults due to caries and excessive consumption of sweets and acidic beverages leads to questions about why there is an increase of cases despite all means of information. Is there a lack of interest in oral health among young adults? The aim of this study was to evaluate the knowledge and skills level of the students regarding oral health in order to conceive and implement an oral health educational program adapted to their needs and interests.

The survey method using a questionnaire was administered to students in the first, second, and third academic year at the “Ferdinand I” Military Technical Academy (Bucharest, Romania) in March 2019 and included general data and specific information about oral health. We noted a relatively high participation in this study (73.91% agreed), although 71% have not participated in campaigns and training programs about oral health. The participants showed a high degree of interest in dental aesthetics, although 59% consider dental caries to be the main problem affecting their oral-dental health. It is imperative to introduce an educational module regarding oral health for military students, and not only, in order to materialize knowledge by adopting healthy attitudes and behaviors, avoiding high financial and biological costs for both the patient and the healthcare system.

## Introduction

The idea of this study started from the need to understand and evaluate the level of knowledge and skills of the students of the Military Technical Academy “Ferdinand I” in order to be able to design and subsequently implement an educational program adapted to the needs and interests of this category of patients, in order to improve the level of oral health by reducing the number of carious lesions, improving oral hygiene indices, and periodontal status, as well as correcting dento-maxillary anomalies [1]. By improving and maintaining an optimal level of oral health, we aim at maintaining the combative capacity of the future military at an appropriate level, an essential aspect, especially in the situation of participating in combat missions in isolated areas, such as the missions in Theaters of operations.

We understood the opportunity of such a program as a result of a professional career of more than ten years, both as a dentist in the Military Technical Academy “Ferdinand I” and as a military doctor, actively participating in two peace missions in the Afghanistan Theater of operations. Case studies encountered in the country, as well as in the Theater of operations, highlighted gaps in terms of education in the field of oral health and skills related to oral cavity hygiene.

From the discussions with the patients, we observed on their behalf a lack of compliance in the use of the auxiliary means of oral hygiene for the removal of the interdental bacterial plaque on the one hand due to the little knowledge about it, and on the other hand due to the lack of motivation in their use (a good part of the patients stating that they do not notice a visible, immediate improvement of oral hygiene). These aspects capture gaps in the health education of the subjects [2].

## Material and Methods

In order to assess the knowledge and skills level on oral health among military students, we built and validated a questionnaire with 22 items, which also reported data on general information about the respondents.

The target population is represented by military students, first (N=327), second (N=259), and third (N=219) year of study, enrolled in military-technical programs at the “Ferdinand I” Military Technical Academy.

The criteria for inclusion in the study were:

• Student status at the “Ferdinand I” Military Technical Academy.
• Filling in the informed consent form for participation in the scientific research study.

Since the answer to the questionnaire is voluntary, with random selection, the sample is one of convenience.

The sample volume for a representative sample was estimated using Open-Epi, a web-based open-source interface [3] under the following hypothesis:

• Population size N= 805;
• Hypothesized proportion for outcome factor in the population p=0.5, while this implies the maximum variance, assuming in this way the highest incertitude;
• Margin of error Δ(p)=+/-5 p.p;
• Assumed type I error = 0.05 - Confidence level 95%.

In these conditions, the estimated sample volume is 261 units (students).

The study was carried out between March 2019 - December 2019 during a meeting organized in Aula Magna of the abovementioned institution. The students were informed about the research, and the ones that gave their informed consent to participate in the scientific research study completed the questionnaire.

The oral health knowledge and skills assessment form comprise two sections:

1. General information
2. Specific information related to oral health

In the general information section, data related to:

• Year of study
• Age
• Gender
• Residence: urban or rural area
• Educational level of parents
• Regarding the specific information related to oral health, three directions were followed:
• Oral cavity hygiene
• Eating habits
• Patient-dentist relationship

The first direction includes a set of nine questions regarding dental brushing, self-assessment of dental brushing, and auxiliary means of oral cavity hygiene.

The second direction comprises a set of three questions related to the consumption of sweet foods and beverages, the consumption of sour beverages, and the possible consumption of food or beverages (except water) after brushing.

The third direction includes a set of ten questions related to patient-dentist relation, namely the existence or absence of an attending dentist for each subject, the frequency of the dental checks, the degree of interest expressed by both the doctor and the patient regarding the education and information on topics related to oral health, the reason and the moment of the last visit to the dentist, possible participation in campaigns to promote oral health.

Besides basic descriptive statistics, differences between the groups were tested using Pearson’s correlation coefficient X2=nΣ[(Oi-Ei)2/Ei], where the Oi and Ei denote the observed and expected shares (%) defined by the counter i. The null hypothesis was rejected if the p-value was less than 0.05.

The association between students’ characteristics and different oral health-related behaviors was analyzed using the Chi-Squared Statistic, Fischer Exact Test, Fischer – Freeman – Halton Exact Test, or Crosstabulation.

For statistical data analysis, we used the IBM SPSS Statistics software, v27. The results are presented below.

## Results

Out of the total of 805 first-year (327), second-year (259) and third-year (219) students, a number of 595 students gave informed consent to participate in the study. Thus, we included 256 first-year students, 193 second-year students, and 146 third-year students. From a percentage point of view, 73.91% of the students invited to participate in the study responded favorably, a representative sample as estimated using the Open-Epi web-based open-source interface. Regarding the distribution by years point, the most receptive were the students from the first year - 78.28%; in the second place were those from the second year - 74.51%, and students from the third year were in the last place, participation being below 70%, namely 66.66%.

Analyzing the study participants, it can be noted that most participants are male: 71.6% (426), have an average age of 20 years: 33.1% (197), come from the urban area: 68.2% (405) and have parents with a high educational level (over 30% have an academic degree).

Most students brush their teeth twice a day (morning and evening): 78.3% (466), and those who opt for a single brush per day, practice this habit mainly in the morning: 16.5% (98). The Fischer-Freeman-Halton Exact Test identified no statistically significant difference between groups by residence (p-value >0.05). However, there was a statistically significant difference between groups by gender (p-value <0.05), females brushing their teeth twice a day in a larger number than expected; instead, males brush their teeth once a day (in the evening or morning) in a larger number than expected.

Regarding the type of brush used, most use a manual toothbrush: 77.5% (461), and a small part combines the manual brush with the electric brush: 3.5% (21). The Chi-Squared test identified no statistically significant difference between groups by gender (p-value >0.05), but there was a statistically significant difference between groups regarding residence (p-value <0.05); the ones that come from urban areas prefer an electric toothbrush or combining manual and electric toothbrushes in a larger number than expected; however, the participants that come from rural areas use a manual toothbrush in a larger number than expected.

More than half of the respondents change their toothbrush every three months: 53.8% (320), and a relatively high percentage: 34.3% (204) even more often - once a month. There is no statistically significant difference between groups by gender, but there is a statistically significant difference (p-value < 0.05) between residences; those from urban areas change their toothbrush every three months in a larger number than expected.

**Figure 1:**
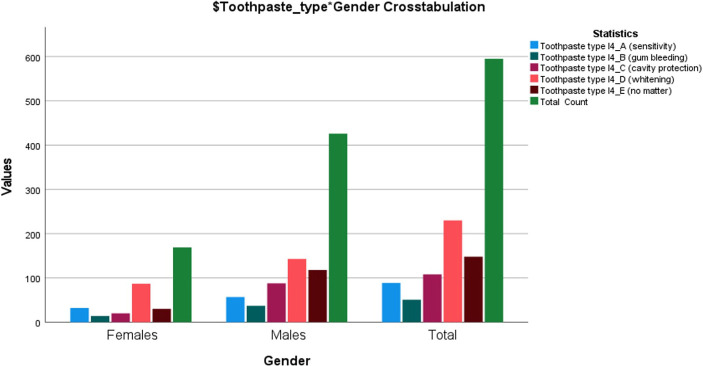
Crosstabulation of gender regarding toothpaste type. The most popular response for both females (51.3%) and males (33.6%) is suggesting the use of whitening toothpaste (Toothpaste type I4_D - whitening) followed by the sensitivity toothpaste (Toothpaste type I4_A - sensitivity) for females (18.9%) and no matter toothpaste (Toothpaste typeI4_E - no matter) for males (27.7%).

Regarding the type of toothpaste used, there is a tendency to choose toothpaste with a whitening effect: 35% (208), at a considerable distance being those who use a toothpaste against tooth decay: 17% (101). The number of those who do not pay attention to the type of toothpaste used is quite significant: 24.9% (148). Crosstabulation by gender and multiple responses set for item 4 section I regarding the toothpaste type used by respondents is presented in Figure 1.

Comparing the data related to the type of toothpaste used with those obtained when asked about the problems affecting oral-dental health, it is observed that although almost 60% of the subjects consider that tooth decay is in the first place, most subjects chose a toothpaste with a whitening effect (35%) or do not pay any attention to the type of paste used (24.9%), the group opting for a toothpaste with protective effects against tooth decay (17%) being only in the third place.

Fluorine content is not a relevant criterion in choosing toothpaste for more than half of the subjects: 52.4% (312), as they do not know whether or not the toothpaste they use contains fluorine. There is no statistically significant difference between groups by gender, nor by residence (p-value >0.05), according to the Fisher-Freeman-Halton Exact Test.

The consumption of toothpaste exceeds three tubes per year for most students - 72.4% (431); almost 20%, namely 18.2% (10), consume three tubes per year, and only a small part - 0.8% - consume one tube per year (5). The Fisher-Freeman-Halton Exact Test suggests there is a statistically significant difference between males and females (p-value<0.05). Males use two or three tubes/year in a larger number than expected (count: 47/84, while females use more than three tubes/year in a larger number than expected (count: 142, expected count: 122.4).

Self-assessment of the way the teeth are brushed brings to light the “good” rating for almost half of the respondents: 48.1% (286) and “very good” for a slightly smaller number of them: 42.5% (253). There is no statistically significant difference between groups by gender, nor by residence (p-value >0.05), according to the Fisher-Freeman-Halton Exact Test.

Most of the studied group allocates three minutes for oral cavity hygiene: 56% (333). In second place are those who allocate two minutes for this activity: 26.4% (157). There is also a percentage worth considering: 12.3% (73), which allocates a significantly longer time for oral cavity hygiene - ten minutes. There is no statistically significant difference between groups by gender, nor by residence (p-value>0.05), according to the Fisher-Freeman-Halton Exact Test.

Water mouthwash is the most widely used auxiliary mean for oral cavity hygiene, both alone - 17.5% (84) and in combination with other auxiliary means: mouth rinse and dental floss - 41.5% (247), mouth rinse, dental floss, and other means - 13.6% (81). However, almost one-fifth of the students - 19.2% (114) do not use any auxiliary means of oral cavity hygiene.

In terms of eating habits, we note the daily presence in the diet of foods containing sugar for most of the respondents - 70.9% (422), and the consumption once a few days is valid for more than 20% of the subjects: 22.5% (134). A small part of the studied group consumes rarely or at all such foods - 2.2% (13) and 0.2% (1). We also noticed a statistically significant difference (p-value <0.05 at the Fisher-Freeman-Halton Exact Test) between females and males. Females consume daily foods containing sugar in a larger count than expected (count: 138, expected count: 120) while men consume foods containing sugar once a few days or less, a larger count than expected (count: 111, expected count: 96).

Half of the respondents regularly consume fizzy drinks (35.6% (212) - once a few days, 15.3% (91) - weekly), while 12.4% (74) are daily consumers and a small part (6.2% (37) does not consume such drinks at all. The Chi-Squared Test suggests a statistically significant difference between groups by gender (p-value <0.05), males consuming more often fizzy drinks than females in a larger count than expected, as suggested in Table 1.

**Table 1:** Gender*II2 Crosstabulation.

					II2			
			A	B	C	D	E	Total
Gender	F	Count	15	53	21	66	14	169
		**Expected Count**	21.0	60.2	25.8	51.4	10.5	169.0
		**% within Gender**	8.9%	31.4%	12.4%	39.1%	8.3%	100.0%
	**M**	Count	59	159	70	115	23	426
		**Expected Count**	53.0	151.8	65.2	129.6	26.5	426.0
		**% within Gender**	13.8%	37.3%	16.4%	27.0%	5.4%	100.0%
Total		Count	74	212	91	181	37	595
	** **	Expected Count	74.0	212.0	91.0	181.0	37.0	595.0
	** **	% within Gender	12.4%	35.6%	15.3%	30.4%	6.2%	100.0%

*II2: How often do you consume fizzy drinks? A – daily; B – once a few days; C – once a week; D – less than once a week; E – never.

The consumption of food or beverages (except water) after brushing occurs regularly in the case of 44.4% (264) of the subjects and several times a week in the case of 26.7% (159) of the respondents. On the other hand, for a small percentage - 9.7% (58), such a situation never occurs. No statistically significant difference was suggested by the Chi-Squared Test (p-value >0.05) between groups by gender or residence.

Regarding the patient-dentist relationship, only slightly more than half of the students declare they have a current dentist - 55.9% (331). The Fisher-Freeman-Halton Exact Test suggests a statistically significant difference between groups by gender and residence (p-value <0.05), females and the participants from urban areas have an attending dentist in a larger count than expected.

A considerable percentage of the respondents - 35.8% (213) perform the dental check-up twice a year, to a lesser extent - 29.1% (173) once a year, and over a fifth - 22.5% (134) see the dentist only when they have dental pain. The Fisher-Freeman-Halton Exact Test suggests no statistically significant difference between groups by gender and residence (p-value >0.05).

An overwhelming majority of the study group considered receiving relevant and sufficient information to improve their oral health - 80.5% (479). Only a small part of the students - 10.8% (64) stated that they received relevant information but insufficient in this regard. There is also a percentage of 7.6% (45) who did not receive any information in this regard from the dentist. The Fisher-Freeman-Halton Exact Test suggests a statistically significant difference (p-value <0.05) in the group that has an attending dentist and receives relevant and sufficient information in a larger count than expected. In comparison, the group that does not have an attending dentist receives less relevant and insufficient information in a larger count than expected. This highlights the efficient and better communication between patients and attending dentists.

The means of information regarding oral and dental health are mainly represented by articles found on the Internet - 39.7% (236) and questions to the dentist - 32.3% (192). Fewer respondents use social media as a means of information in this regard - 12.3% (73). There is also a small percentage - 5.2% (31) that was not documented in any way on this subject.

The main problems affecting the oral-dental health of the studied group are mainly represented by dental caries - 58.8% (350) of the subjects, gingival problems - 10.4% (62) of the subjects, and incorrect positioning of the teeth - 8.4% (50) of the subjects. On the other hand, almost one-fifth of the students -19.5% (116), do not consider that they have a problem that affects their dental health.

Regarding basic knowledge in the field of biology, most of the respondents know the number of teeth a man has in his life - 82% (487): two sets of teeth, as well as the number of teeth that an adult has - 94.1% (566): 32 teeth. The Fisher-Freeman-Halton Exact Test suggests there is a statistically significant difference between groups by gender (p-value <0.05); females give in a larger count than expected the correct response regarding the number of sets of teeth, but there was no statistically significant difference between groups by gender (p-value >0.05) concerning the response to the question that refers to the number of teeth that an adult has.

The last visit to the dentist was a few months ago for 44.4% (264) of the subjects and a year ago for 22.2% (132). On a different note, there is a category of patients who do not remember when they last saw at the dentist - 15% (89).

The main reason for the last visit to the dentist is a preventive check-up for 36.1% (215) of students, followed by dental pain for a slightly smaller number of students - 32.9% (196). At the same time, almost a quarter of the studied group last saw the dentist for a reason other than dental pain, preventive check-up, gingival bleeding, inflammation, or dental trauma. The Fisher-Freeman-Halton Exact Test suggests no statistically significant difference between groups by gender and residence (p-value >0.05).

Most respondents stated that they had not participated in or received information regarding oral health promotion campaigns - 70.6% (420), while 29.4% (175) gave an affirmative answer in this regard. Fisher’s Exact Test suggests no statistically significant difference between groups by gender and residence (p-value >0.05).

## Discussion

The availability of students to participate in this study was relatively high.

Although most of the respondents respect the general instructions regarding the hygiene at the gold-dental level - to brush their teeth both in the morning and in the evening for three minutes, they change their toothbrush once every three months and consume on average over three tubes of toothpaste a year, almost half of them consider that they wash their teeth “well” (not “very well” or “excellent”).

The auxiliary means of hygiene of the oral cavity is mainly limited to water mouthwash, whether or not associated with other auxiliary means, such as dental floss. There is a considerable percentage of subjects who do not use any auxiliary means for oral cavity hygiene.

The interest expressed by the respondents regarding the toothpaste used is in the sense of satisfying their desire to have teeth as white as possible, so they often choose a toothpaste with a whitening effect.

Most of the respondents consume sugar-containing foods daily and sour drinks periodically. The conditions associated with the prolonged consumption of these drinks manifest themselves both locally through dental wear and tear, which are often grafted on a particular caries pattern, as well as in general: type 2 diabetes[4-6], myocardial infarction [7, 8], gout [9, 10], obesity and obesity-associated diseases[11], and bone damage [12, 13].

According to the TNS Opinion & Social (2010) report on Oral Health, the percentage of those who went to the dentist for a dental check-up is lower than the European average: 36.1% versus 50%, respectively [14]. The percentage of those who went to the dentist less than a year ago exceeds that of the European average: 66.6% versus 57%, respectively [15]. Almost double the number of patients presented to the dentist for a dental emergency compared to the EU average, a percentage that is closer to the average in Romania: 32.9% compared to 17% the European average and 40% the Romanian average [15]. The primary means of information on topics related to oral health are represented by the online environment (through articles found on the Internet and social media) and the questions addressed to the dentist, the information received from the latter being of real use (both sufficient and relevant) to the greatest extent. The main health problem at the oro-dental level is represented by dental caries, despite the favorable elements of good oral hygiene (information on topics related to oral health, dental check-ups, correct hygiene) that results from the students’ statements. However, the interest shown by the respondents regarding oral cavity care mainly concerns issues related to dental aesthetics, the functionality related elements remaining in the second place.

In the absence of a national program of health education of the population regarding oral health, the damage of the dento-maxillary apparatus appears at an increasing age with increasingly complex treatment needs [16] and high biological and financial costs for the patient and the health system [17].

## Conclusions

We consider that it is imperative to introduce an educational module regarding oral health in the training program for military students, and not only.

Information given to students must be correlated to the non-parametric statistical tests results to make up for educational shortcomings and correct some oral health harmful behavior.

Starting from the results obtained by processing the questionnaires administered to the students participating in this study, the central idea of such a module is to improve the level of knowledge regarding oral health by using the teachers and dentists in the educational institutions as promoters [18]. This knowledge will be materialized by adopting healthy attitudes regarding oral-dental care with both personal (as a potential patient) and professional implications (as a future officer/military, potentially participating in military missions in conflict areas, isolated, where access to specialized healthcare is difficult, maybe even impossible in certain situations or periods).

## Conflict of Interest

The authors declare that there is no conflict of interest.
